# CXCR4 Knockdown Via CRISPR/CAS9 in a Tumor-Associated Macrophage Model Decreases Human Breast Cancer Cell Migration

**DOI:** 10.7759/cureus.20842

**Published:** 2021-12-30

**Authors:** Luis Jaramillo-Valverde, Kelly S Levano, Silvia Capristano, David D Tarazona, Alberto Cisneros, Velia M Yufra-Picardo, Julio Valdivia-Silva, Heinner Guio

**Affiliations:** 1 Biotechnology and Molecular Biology Laboratory, Instituto Nacional de Salud, Lima, PER; 2 Faculty of Health Sciences, Universidad de Huánuco, Huánuco, PER; 3 Cell Culture Laboratory, Instituto Nacional de Salud, Lima, PER; 4 Innóvate Peru, Ministerio de la Producción, Lima, PER; 5 Bioengineering Department, Universidad de Ingenieria y Tecnologia - UTEC, Lima, PER; 6 Graduate School, Universidad Científica del Sur, Lima, PER

**Keywords:** breast cancer, cxcr4, metastasis, macrophages, genomic editing

## Abstract

Introduction

Breast cancer is the leading cause of cancer-related deaths in women worldwide with the majority of deaths due to metastasis. The development of metastasis is closely related to the tumor microenvironment where tumor-associated macrophages (TAMs) are the main immune cell component playing a crucial role in tumor migration. Key players in tumor progression, metastasis and survival are the receptor CXCR4 and its ligand CXCL12. CXCR4 is expressed in multiple cell types including macrophages and breast cancer cells. Many studies have focus on the role of CXCR4 expressed in breast cancer cells.

Methods

In this study, we investigated the role of CXCR4 expressed in TAMs on breast cancer cell migration by reducing CXCR4 expression via CRISPR-CAS9 system in differentiated THP-1 cells (a TAMs model).

Results

According to wound healing migration assay, MCF7 cancer cells co-cultured with genetically edited dTHP-1 cells have a lower migration rate as compared to MCF7 cancer cells co-cultured with unedited and dTHP-1 cells.

Conclusion

The study demonstrates the role of CXCR4 on breast cancer cell migration through TAM-cancer cell crosstalk.

## Introduction

Metastasis is the main cause of death in patients with breast cancer; therefore, it is essential to prevent metastasis by inhibiting its early development. When cancer occurs, the immune system intervenes by detecting transformed cells. Usually, immune cells detect, attack and eliminate transformed cells. However, studies have shown macrophages with a pro-tumorigenic phenotype [1,2] called TAMs (tumor-associated macrophages). The chemokine CXCL12 and its receptor, CXCR4 and CXCR7, play an important role in breast cancer growth and metastasis [3-5]. Metastasis results in multiple processes, starting with the escape of cancer cells from the primary tumor. TAMs work on primary tumor-promoting invasion, migration, extravasation and suppression of immune response. These processes are important for metastasis onset. TAMs interact with a wide range of growth factors, cytokines and chemokines in tumor microenvironment [2].

There is evidence that cancer cells and leukocytes contain high levels of CXCR4 receptor expression [6,7]. CXCL12 activates multiple genes including regulated extracellular kinase 2 (ERK2), protein kinase B (PKB), phosphatidylinositol 3-kinase (PI3K), steroid receptor coactivator (SRC) and mitogen-activated protein kinase (MAPK), resulting in the regulation of cell growth, survival and cancer metastasis [8,9].

TAMs presence in blood has been linked to poor prognosis in breast cancer [10], ovarian cancer [11], glioma types [12] and lymphoma [13], and to better prognosis in colon and stomach cancers [14] and incongruent results in lung cancers [15,16] and prostate cancers [17]. It has been shown that TAMs differ in their functions, depending on the tumor type with which they are associated [18].

The role of CXCR4 expression on breast cancer cells has been extensively studied. However, in this study, we demonstrate that there is also a role of CXCR4 on breast cancer cell migration when it is expressed on TAMs. CXCR4 can be a key regulator in the cross-talk between breast cancer cells and TAMs promoting migration.

## Materials and methods

Cell culture

In this study, we used the cell lines: THP-1 leukemic monocyte cell line (TIB-202) and MCF-7 human breast cancer cell line (HTB-22). They were obtained from ATCC and cultured using RPMI with 10% Fetal Bovine Serum (FBS). For THP-1 cells, 2-mercaptoethanol (0.05mM) was added to the cultured media.

CRISPR/Cas9-mediated knockdown of CXCR4 in THP-1 cells

To generate CXCR4 knockdown on THP-1 cells via the CRISPR/Cas 9 genome editing system, the GeneArt CRISPR Nuclease Vector with OFP Reporter Kit (Life Technologies, Carlsbad, CA) was used. The target-specific oligonucleotides used were: Top strand oligo 5'-CACCGCATCTGGAGAACCAGGTTTT-3' and bottom strand oligo 5'-CTGGTTCTCCAGATGCGGTGCGGTG-3'. They were ligated into the GeneArt CRISPR Nuclease Vector following the manufacturer’s instructions. The presence of the double-stranded oligonucleotide insert in positive transformants was confirmed by Sanger sequencing.

Undifferentiated THP-1 cells cultured in suspension were transfected with the CRISPR/Cas9 construct and Lipofectamine 2000 (Life Technologies) in 6 well plates under serum-free media conditions. Two sets of transfected cells were generated: (1) for migration assay experiments and (2) for real-time quantitative polymerase chain reaction (RT-qPCR) experiments. Fetal Bovine Serum (FBS) was added three hours post transfection. Cells were differentiated after 24 hours using phorbol 12-myristate 13-acetate (PMA) in complete media. To check for mutated THP-1 cells, GeneArt Genomic Cleavage Detection Kit (Life Technologies) was used according to manufacturer’s instructions.

Co-culturing

Forty-eight hours after transfection, the PMA-containing medium was removed, the differentiated THP-1 (dTHP-1) cells were washed with PBS (Phosphate buffered Saline) and new medium with 1% FBS was added to the cells. MCF7 cells were added and allowed to attach to the wells.

Pro-migration assay

To examine migration, we used the wound healing migration assay. MCF7 cells were co-cultured with dTHP-1 cells for 24hrs. After incubation, the cells were scratched in a line across the well with a 10ul pipette tip. The cells were then washed to remove cell debris. Images of the same location of a cell-free wound were recorded from 0-48hrs. Cell migration was assessed using free Image J software (version 1.50i, NIH).

RT-qPCR

Analysis of the MAPK signaling enzymes (p44/42 MAPK and PI3K) mRNA levels was performed using RT-qPCR. After coculturing for 24hrs, total RNA was extracted from MCF7 cell using RNeasy Protect Mini Kit (Qiagen, Hilden, Germany). Reverse transcription was performed using SuperScript First Strand Synthesis System (Invitrogen, Waltham, MA) according to the manufacturer’s protocol. Random primers were used. qPCR was performed on a Rotor Gene 2000 (Corbett Research, Sydney, Australia) using the KAPA SYBR FAST qPCR Kit Master Mix (Kapa Biosystems, Wilmington, MA) and target-specific primers (Table 1). Results were normalized with GAPDH.

**Table 1:** The primers and reaction conditions for real-time PCR

Gene	Primer	Ta (C°)	Production (bp)
p38MAPK	Forward: CATGGCGGATGACCTAAAGC	55	186
	Reverse: TTGCTTCCTTGGTCTGTTGC		
PIK3CA	Forward: CCAGACCAGTACGTTCGAGA	55	178
	Reverse: GAAACTGCCCTATCCTCCGA		

Statistical analysis

Each trial was repeated at least three times, and the data were expressed as the mean. The data were compared between groups by independent sample t-test and paired sample t-test. Data analysis was performed using Stata v15 (StataCorp, College Station, TX) considering a statistical significance of p < 0.05.

## Results

The human leukemic monocyte cell line THP-1 was used as a model for TAM. The monocyte cell line was differentiated into macrophages with PMA. Expression of CXCR4 by THP-1 cells was confirmed by RT-qPCR (data not shown). The knockdown expression of CXCR4 was achieved by CRIPSR-CAS9 system; we designed sgRNAs targeting CXCR4 exon 2. sgRNA target sites were selected for minimal predicted off-target activity and maximal on-target activity, according to established algorithms (using Benchling).

Confirmation of CXCR4 knockdown by RT-qPCR

**Figure 1:**
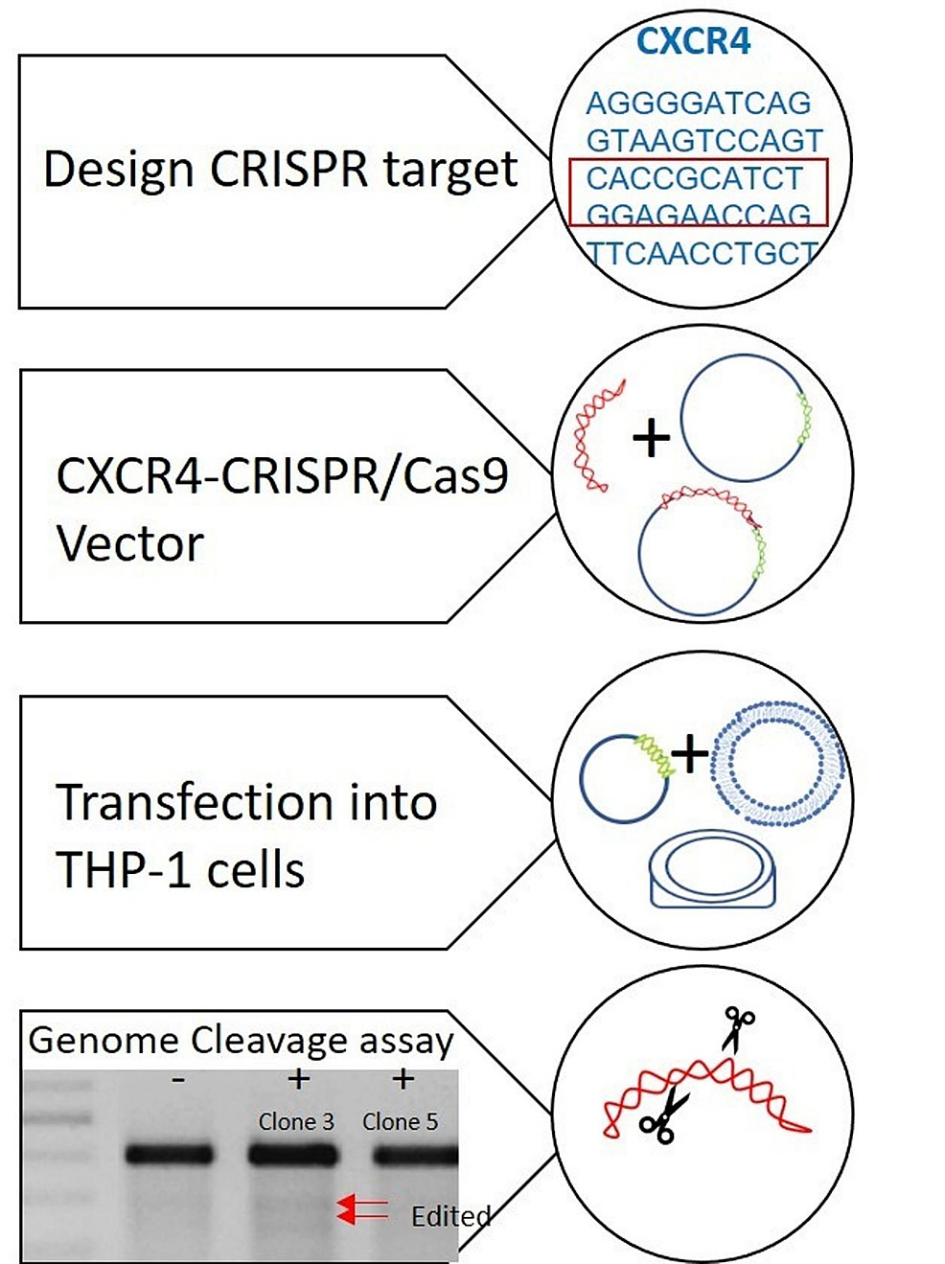
CRISPR/Cas9-mediated gene targeting of CXCR4 in THP-1 cells. Schematic diagram of gene targeting steps ending with genome cleavage assay for mutation detection in THP-1 cells Image credits: Kelly S. Levano

Two positive clones selected by genomic cleavage detection kit (Life Technologies) and sequencing were confirmed by RT-qPCR to ensure the absence of CXCR4 mRNA expression and therefore successful knockdown of CXCR4 gene (Figure 1). All confirmed clones were used for downstream experiments.

Effects of CXCR4 KO on MAPK signaling pathway

**Figure 2:**
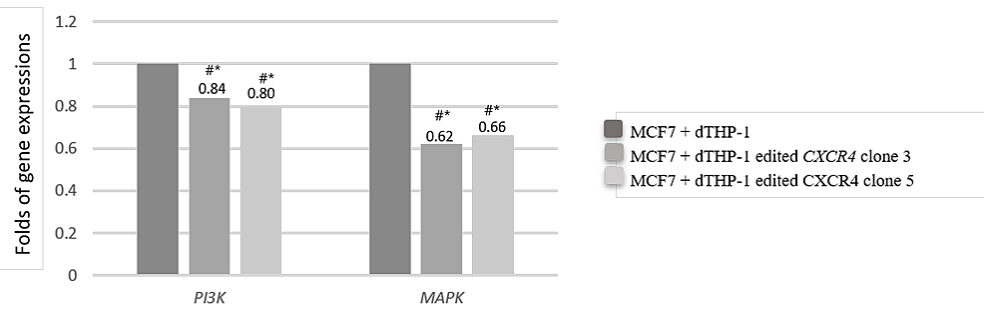
mRNA expression levels of PI3K and MAPK in MCF7 cells co-culture with dTHP-1 transfected with edited CXCR4 Symbol “#” represents the comparison with the no edited CXCR4 group (*p<0.001)

We next assessed the effect of CXCR4 knockdown expression in THP-1 macrophage cells on the activation of oncogenic MAPK pathway in breast cancer cells (MCF7). RT-qPCR confirmed a significant reduction in the levels of p44/42 MAPK and PI3K showing the importance of receptor CXCR4 on breast cancer cell growth (Figure 2).

CXCR4 knockdown in THP-1 macrophage cells decreases migration of MCF7 breast cancer cells

**Figure 3:**
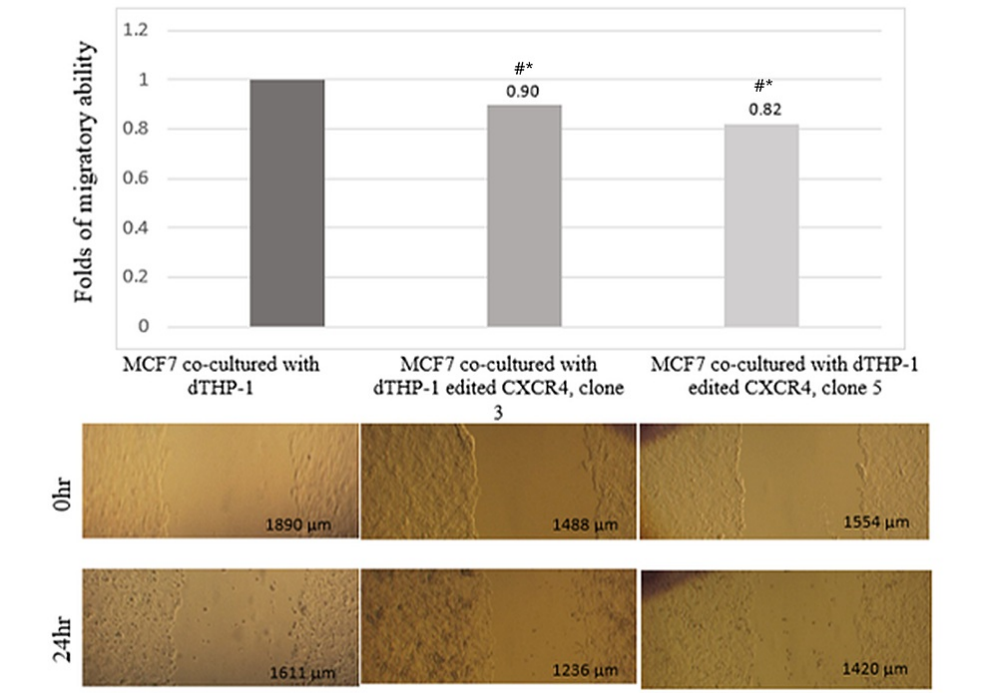
Wound healing migration assay of MCF7 cells co-culture with dTHP-1 transfected with edited CXCR4 A: MCF7 + dTHP-1. B: MCF7 + dTHP-1 edited CXCR4 clone 3. C: MCF7 + dTHP-1 edited CXCR4 clone 5. Symbol “#” represents the comparison with the no edited CXCR4 group (*p<0.001)

After demonstrating activation of the oncogenic MAPK pathway by knocking down CXCR4 expression, we investigated phenotypic changes in MCF7 breast cancer cells co-cultivated with THP-1 cells expressing reduced levels of CXCR4. We used the wound healing assay to access the potential of MCF7 cells to migrate. According to wound healing migration assay, MCF7 cancer cells co-cultured with genetically edited dTHP-1 cells have a lower migration rate as compared to MCF7 cancer cells co-cultured with unedited and dTHP-1 cells (Figure 3).

## Discussion

Several studies report that inflammatory response perturbations increase pathogenesis likelihood in diseases related to metabolism, autoimmunity and malignant transformation [19-21]. Breast cancer, for example, is characterized by an elevated secretion of proinflammatory cytokines (IL-1, TNF-α, IL-6) or anti-inflammatory cytokines (IL-10), chemokines and chemokine receptors (CXCL8, CXCR4), and angiogenic factors (VEGF) [21]. This network of proinflammatory components competes with inhibitory effect of anti-inflammatory cytokines (IL-4) on breast cancer cells growth [22] and promotes cell proliferation, tumorigenesis and metastasis [19]. Therefore, it is clear that the study of the expression level of chemokines and chemokine receptors activity is important to understand the pathways that promote cancer progression.

The clinical data also showed that high expression levels of CXCL12 and its receptors CXCR4 were associated with easy metastasis and poor prognosis of breast cancer [23]. Some studies have also shown that CXCR4 and CXCR7 promote cell migration, invasion, angiogenesis, and tumorigenesis through different modes of signal transduction based on their respective characteristics [24,25].

Chemokines and their receptors are very important to the progress of tumors [26]. There are many reports on the effect of CXCR4 gene silencing on tumor biological characteristics. For example, silencing the CXCR4 gene in the breast cancer cell line can significantly inhibit the growth of tumor cells, reduce colony formation, and increase the sensitivity of cisplatin chemotherapy [27], and CXCR4 gene silencing can significantly block the metastasis of breast cancer [28].

In this work, genetic expression of CXCR4 on THP-1 cells was evaluated by real-time qPCR. In agreement with previous studies, our results show high levels of CXCR4 mRNA in dTHP-1 cells [29]. We were able to knockdown the expression of CXCR4 by editing genetically THP-1 cells with the CRISPR-CAS9 system. Our work further demonstrates that the receptor CXCR4 expressed in macrophages is important for breast cancer cell growth and migration. Reduction of CXCR4 expression decreases the mRNA levels of PI3K and MAPK.

Furthermore, migration was also affected with decrease expression of CXCR4. Our findings suggest that the modification induced in the CXCR4 gene of TAM cells repressed the levels of migration speed in MCF7 cells, inhibiting the metastasis cascade for this cell type. This result is similar to that found in decreased migration of lung alveolar epithelial cells CXCR4 knockout mediated [30].

With these preliminary results, we can further study the specific signal pathways and mechanisms that affect TNBC after CXCR4 knockout and the mechanism of inhibition of breast cancer progress after single knockout, which may provide a new target for the treatment of breast cancer. Evaluation of other cancer cell lines for coculture will allow us to cover different cell models with different metastasis rates.

This study has some limitations. The small six-sample size might intervene with the findings. In addition, we didn't evaluate the model in human cells, only in one breast cancer cell line. Finally, we recommend further studies to determine the integrated effects of CXCL12, CXCR4, and CXCR7 on host immune responses to breast cancer.

## Conclusions

Our study confirms the important role of receptor CXCR4 on breast cancer cell growth and migration. We were able to knockdown the expression of CXCR4 by editing genetically THP-1 cells with the CRISPR-CAS9 system and demonstrate that the reduction of CXCR4 expression decreases the mRNA levels of PI3K and p38MAPK. We showed that this effect is achieved through the cross-talk between macrophages and breast cancer cell lines, where expression of CXCR4 in macrophages appears to be crucial for breast cancer cell migration. These results suggest that CXCR4 is involved in the progression of breast cancer, and deserve further investigation to determine its potential as a new target for breast cancer therapy.
